# Multistate nontyphoidal *Salmonella* and Shiga toxin-producing *Escherichia coli* outbreaks linked to international travel—United States, 2017–2020

**DOI:** 10.1017/S0950268823002017

**Published:** 2024-01-11

**Authors:** Caroline A. Habrun, Meseret G. Birhane, Louise K. François Watkins, Katharine Benedict, Lyndsay Bottichio, Kaylea Nemechek, Beth Tolar, Morgan N. Schroeder, Jessica C. Chen, Hayat Caidi, Misha Robyn, Megin Nichols

**Affiliations:** 1 Division of Foodborne, Waterborne and Environmental Diseases, National Center for Emerging and Zoonotic Infectious Diseases, Centers for Disease Control and Prevention, Atlanta, GA, USA; 2 Centers for Disease Control and Prevention, Epidemic Intelligence Service Program, Atlanta, GA, USA; 3 Oak Ridge Institute for Science and Education, Oak Ridge, TN, USA

**Keywords:** international travel, outbreak, *Salmonella*, STEC, travel-associated, antimicrobial resistance

## Abstract

Enteric bacterial infections are common among people who travel internationally. During 2017–2020, the Centers for Disease Control and Prevention investigated 41 multistate outbreaks of nontyphoidal *Salmonella* and Shiga toxin-producing *Escherichia coli* linked to international travel. Resistance to one or more antimicrobial agents was detected in at least 10% of isolates in 16 of 30 (53%) nontyphoidal *Salmonella* outbreaks and 8 of 11 (73%) Shiga toxin-producing *E. coli* outbreaks evaluated by the National Antimicrobial Resistance Monitoring System. At least 10% of the isolates in 14 nontyphoidal *Salmonella* outbreaks conferred resistance to one or more of the clinically significant antimicrobials used in human medicine. This report describes the epidemiology and antimicrobial resistance patterns of these travel-associated multistate outbreaks. Investigating illnesses among returned travellers and collaboration with international partners could result in the implementation of public health interventions to improve hygiene practices and food safety standards and to prevent illness and spread of multidrug-resistant organisms domestically and internationally.

## Key results

- Returning US travellers are acquiring enteric bacterial infections from travel to international locations; CDC investigated 41 travel-associated nontyphoidal *Salmonella* and Shiga toxin-producing *Escherichia coli* outbreaks and1,066 illness during 2017–2020.
- Travel-associated outbreaks were commonly associated with popular international destinations for US travellers.
- Fifty-nine per cent of travel-associated outbreaks contained ≥10% isolates that conferred resistance to one or more drugs tested by the National Antimicrobial Resistance Monitoring System.

## Introduction

The number of international travellers from the United States rose yearly until the COVID-19 pandemic began in 2020. International travel reached an all-time high in 2019 with 99 million people travelling internationally from the United States, an increase from 93 million in the previous year [1]. Notably, gastrointestinal disease is the most reported illness in returning travellers [2]. From 2017 to 2019, the Foodborne Diseases Active Surveillance Network (FoodNet) reported that 14% of enteric bacterial and parasitic infections were linked to international travel, although federal travel restrictions reduced this to 5% in 2020 [3]. Travel-associated bacterial illnesses were most frequently caused by *Campylobacter*, *Salmonella*, Shiga toxin-producing *Escherichia coli* (STEC), and *Shigella*, which can be transmitted by contaminated food and water or contact with infected animals or people [4–6].

Each year, approximately 154 million nontyphoidal *Salmonella* and 2.5 million STEC infections are reported globally [7]. It is estimated that in the United States, approximately 4% of STEC O157 infections, 18% of STEC non-O157 infections, and 11% of nontyphoidal *Salmonella* infections that occur annually are attributable to international travel [8].

Globalization and the rise in international travel and tourism provide the opportunity for antimicrobial-resistant bacteria to spread between geographic areas. [9] Antimicrobial-resistant infections associated with travel have increased over time [10]. Reports of US travel-associated outbreaks often describe a single event. Therefore, the general understanding of the epidemiology and antimicrobial resistance (AR) patterns associated with illness outbreaks among US residents linked to international travel over time remains unclear. This information might be valuable to better inform public health prevention planning, interventions, and messaging regarding illness and international travel. We aim to describe demographics of people becoming ill with nontyphoidal *Salmonella* and STEC infections during or after recently returning from international travel, travel destinations, pathogen serotypes and serogroups, and AR patterns associated with these travel-associated outbreaks affecting residents of multiple states (multistate).

## Methods

### Outbreak definition and detection

We evaluated multistate outbreaks detected by PulseNet, the national molecular subtyping network for foodborne disease surveillance. We analysed data recorded in the Outbreak Management System (OMS), an internal Centers for Disease Control and Prevention (CDC) database for the management of multistate enteric disease outbreak investigations, and the System for Enteric Disease Response, Investigation, and Coordination (SEDRIC), a web-based platform for data sharing and management of outbreak investigations. We defined outbreak strains as a group of nontyphoidal *Salmonella* or STEC isolates highly related by whole-genome sequencing (WGS) [11], indistinguishable by pulsed-field gel electrophoresis (PFGE), or both. Serotype or serogroup of sequenced isolates was confirmed using SeqSero2 (https://github.com/denglab/SeqSero2).

Federal, state, and local health departments using PulseNet routinely monitor for possible multistate outbreaks which are identified as a group of isolates from a single outbreak strain with a suspected common source (e.g., temporal, geographic, travel, or dietary) [12]. Public health officials collect epidemiological data from patient interviews utilizing enteric illness questionnaires, then enter the epidemiological data into OMS and SEDRIC. We used these data to identify outbreaks matching the established definition for travel-associated outbreaks. For this analysis, we defined a travel-associated multistate outbreak as an outbreak that was classified as a possible or confirmed multistate outbreak, involving two or more ill people infected by the same outbreak strain, living in two or more states, with 25% or more of patients with available epidemiological information reporting international travel in the 7 days before illness onset.

### Inclusion criteria

For this analysis, travel-associated outbreaks and the term ‘outbreak’ include all possible and confirmed multistate outbreaks [12] that met the travel-associated outbreak criteria. We included all travel-associated outbreaks reported to and closed by CDC between January 1, 2017, and December 31, 2020, including two outbreaks investigated in 2017, with some people reporting illness onset dates before January 1, 2017, but were not identified and reported to CDC until after January 1, 2017.

### Exclusion criteria

We excluded investigations if less than 25% of patients with available epidemiological information reported international travel in the 7 days prior to illness onset or if all travellers resided in a single state. We also excluded all nonhuman (e.g., food and environmental) isolates from the analysis.

### Descriptive analysis

We summarized multistate outbreak characteristics describing the number of nontyphoidal *Salmonella* and STEC outbreaks per year and the range and median size of each outbreak by pathogen. Additionally, we characterized outbreaks byserotype (s), and the season outbreaks began based on the month of onset of the first reported illness in the outbreak. If illness onset date was unavailable, it was estimated as 3 days prior to specimen collection date. We describedregion (s) and/orcountry (ies) of travel reported by ill people and serotypes associated with each region of travel. Isolates with missing serotypes or those with serotypes differing from the predominant outbreak serotype were further evaluated through WGS or PFGE and determined to be consistent with the predominant outbreak serotype.

We described patient characteristics including proportion of ill people associated with multistate outbreaks by pathogen (e.g., nontyphoidal *Salmonella* and STEC), state of residence, source of infection (e.g., urine, stool, blood, or other), patient demographics (e.g., sex and age), health outcome (e.g., hospitalizations and deaths), multidrug resistance (MDR) related to health outcomes, and region or country of travel. When patient characteristics were unknown or not documented in SEDRIC, they were considered missing. Analysis and data visualization were conducted using SAS version 9.4 (SAS Institute, Cary, NC) or Microsoft Excel.

### Analysis of AR

The National Antimicrobial Resistance Monitoring System (NARMS) evaluated AR for the clinical isolates. Since 2003, the CDC’s NARMS laboratory has performed antimicrobial susceptibility testing (AST) on clinical isolates from outbreak investigations using broth microdilution (Thermo Scientific™ Sensititre™, Westlake, OH) as previously described [13]. The CDC encouraged health departments to submit three or more representative nontyphoidal *Salmonella* or STEC isolates from each enteric bacterial outbreak for AST and results were included in the analysis when available. Drugs tested by NARMS for nontyphoidal *Salmonella* and STEC included amoxicillin-clavulanic acid, ampicillin, azithromycin, cefoxitin, ceftriaxone, chloramphenicol, ciprofloxacin, colistin, gentamicin, meropenem, nalidixic acid, streptomycin, sulfisoxazole, tetracycline, and trimethoprim-sulfamethoxazole. Resistance and intermediate interpretation were determined according to the Clinical and Laboratory Standards Institute (CLSI) breakpoints when available [14]; otherwise, we used NARMS-established breakpoints [15].

Additionally, NARMS screened whole-genome assemblies from clinical enteric pathogen isolates in CDC’s surveillance databases for resistance determinants using an in-house workflow based on ResFinder [16]. De novo assemblies from sequenced isolates were produced using shovill v.1.1.0 (https://github.com/tseemann/shovill). Assemblies were screened for resistance determinants using the ResFinder database (90% identity, 50% cutoff) (updated February 4, 2022) and the PointFinder scheme (updated February 10, 2022) for *Salmonella* spp. implemented in staramr v.0.7.2. Point mutations in *Escherichia* were identified using the PointFinder Scheme for *Escherichia* (updated July 02, 2019), implemented in ARIBA v. 2.12.0 (https://github.com/sanger-pathogens/ariba). WGS can accurately predict resistance based on the presence of resistance determinants in *Salmonella* and *E. coli* isolates [17, 18]. We used AST results to determine resistance when available; when an isolate did not have AST results (or when a drug was not included in the NARMS panel), we predicted resistance based on genes and mutations in the isolate genome known to confer resistance in *Salmonella* or STEC isolates.

For this analysis, we considered an isolate to be resistant to ciprofloxacin if the isolate had a nonsusceptible interpretation by AST or (if no AST results were available) if the isolate carried one or more resistance mechanisms conferring resistance to ciprofloxacin. A single resistance mechanism might result in intermediate (*Salmonella*) or susceptible (STEC) interpretation to ciprofloxacin by AST.

MDR was defined as resistance to at least one drug from three or more antibiotic classes as defined by the CLSI [19]. We defined clinically significant resistance for *Salmonella* isolates as resistance to one or more antimicrobials recommended for empiric or alternative treatment: ampicillin, azithromycin, ceftriaxone, ciprofloxacin, or trimethoprim-sulfamethoxazole [20, 21]. We did not consider STEC isolates to have clinically significant resistance because antimicrobials are not typically recommended for patients with STEC infections. We considered outbreaks to be associated with any resistance, MDR, or clinically significant resistance if ≥10% of the isolates with resistance information met those criteria.

## Results

### Outbreak detection

**Figure 1.**
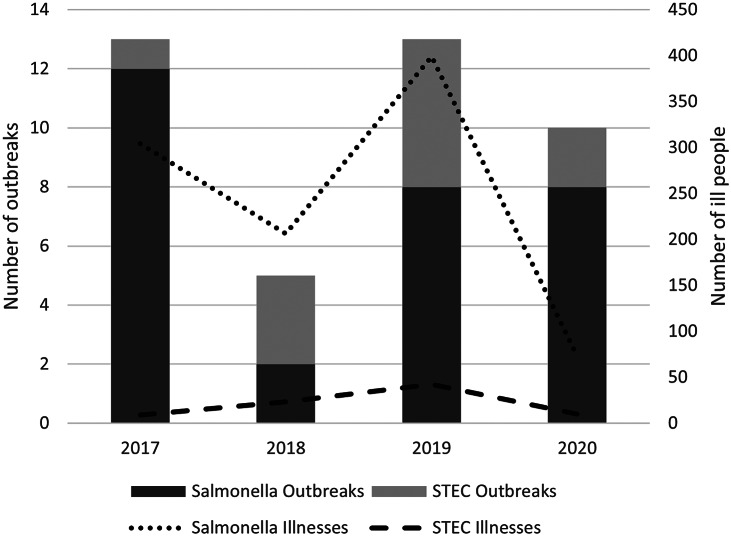
Number of travel-associated multistate outbreaks and number of ill people by pathogen per year—United States, 2017–2020.

**Figure 2.**
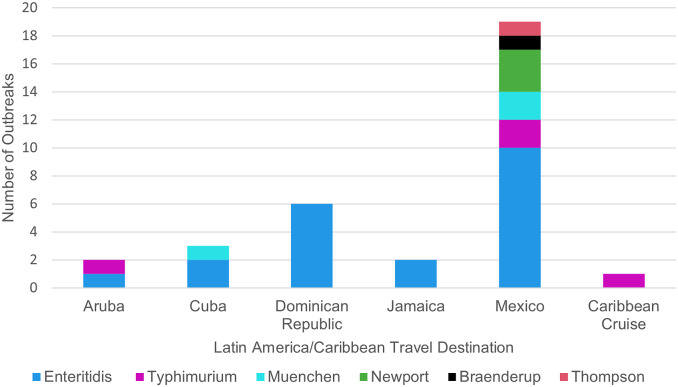
Proportion of multistate travel-associated nontyphoidal *Salmonella* outbreaks and serotypes by destination of travel in Latin America/Caribbean prior to illness onset—United States, 2017–2020. Five *Salmonella* outbreaks consisted of people reporting travel to two Latin America/Caribbean travel destinations prior to illness onset or people in the outbreak reporting travel to different Latin America/Caribbean travel destinations.

During 2017–2020, CDC investigated 30 nontyphoidal *Salmonella* and 11 STEC multistate outbreaks linked to international travel (Figure 1, Table 1). In total,1,066 illnesses occurred as part of the 41 outbreaks among residents of 49 states and the District of Columbia (Table 2). Of the 41 outbreaks, 38 (93%) had 50% or more ill people reporting international travel prior to illness onset. These *Salmonella* outbreaks represented eight serotypes (Braenderup, Concord, Enteritidis, Muenchen, Newport, Strathcona, Thompson, and Typhimurium) and were associated with travel to multiple geographic regions (Table 3). Most nontyphoidal *Salmonella* outbreaks were linked to travel destinations in Latin America/Caribbean (Figure 2). The 11 STEC outbreaks included four serotypes (O111, O157, O103, and O123/O186) and were all associated with travel to Mexico. Nontyphoidal *Salmonella* outbreaks ranged in size from 4 to 365 ill people (median: 13 ill people) and STEC outbreaks from 5 to 12 ill people (median: 7 ill people). Outbreaks of nontyphoidal *Salmonella* tended to occur year-round, while STEC outbreaks typically began in winter or spring (Table 1).

**Table 1.** Proportion of nontyphoidal *Salmonella* and Shiga toxin-producing *Escherichia coli* multistate illness outbreaks including seasonality and region of travel by year and pathogen—United States, 2017–2020

	2017^a^	2018	2019	2020	Total
Characteristics	*n* (%)	*n* (%)	*n* (%)	*n* (%)	*n* (%)
**Outbreak characteristics**					
Outbreaks *n* ^b^	13 (31.7)	5 (12.2)	13 (31.7)	10 (24.4)	41 (100)
**Nontyphoidal** ***Salmonella***	12 (29.3)	2 (4.9)	8 (19.5)	8 (19.5)	30 (73.2)
Seasonality					
Winter (December to February)	3 (10.0)	0 (0.0)	1 (3.3)	4 (13.3)	8 (26.7)
Spring (March to May)	4 (13.3)	2 (6.7)	1 (3.3)	0 (0.0)	7 (23.3)
Summer (June to August)	2 (6.7)	0 (0.0)	3 (10.0)	0 (0.0)	5 (16.7)
Fall (September to November)	3 (10.0)	0 (0.0)	3 (10.0)	4 (13.3)	10 (33.3)
Region of travel^c^^,^^d^					
Asia	2 (6.7)	0 (0.0)	1 (3.3)	0 (0.0)	3 (10.0)
Thailand	1 (3.3)	0 (0.0)	0 (0.0)	0 (0.0)	1 (3.3)
Philippines	2 (6.7)	0 (0.0)	1 (3.3)	0 (0.0)	3 (10.0)
Canada	0 (0.0)	1 (3.3)	0 (0.0)	0 (0.0)	1 (3.3)
Latin America/Caribbean	12 (40.0)	1 (3.3)	7 (23.3)	8 (26.7)	28 (93.3)
Aruba	2 (6.7)	0 (0.0)	0 (0.0)	0 (0.0)	2 (6.7)
Cuba	3 (10.0)	0 (0.0)	0 (0.0)	0 (0.0)	3 (10.0)
Dominican Republic	5 (16.6)	0 (0.0)	1 (3.3)	0 (0.0)	6 (20.0)
Jamaica	0 (0.0)	0 (0.0)	0 (0.0)	2 (6.7)	2 (6.7)
Mexico	6 (20.0)	1 (3.3)	6 (20.0)	6 (20.0)	19 (63.3)
Caribbean cruise	1 (3.3)	0 (0.0)	0 (0.0)	0 (0.0)	1 (3.3)
Europe	0 (0.0)	1 (3.3)	2 (6.7)	0 (0.0)	3 (10.0)
Denmark	0 (0.0)	0 (0.0)	1 (3.3)	0 (0.0)	1 (3.3)
France	0 (0.0)	1 (3.3)	1 (3.3)	0 (0.0)	2 (6.7)
Germany	0 (0.0)	0 (0.0)	1 (3.3)	0 (0.0)	1 (3.3)
Italy	0 (0.0)	0 (0.0)	1 (3.3)	0 (0.0)	1 (3.3)
Spain	0 (0.0)	0 (0.0)	1 (3.3)	0 (0.0)	1 (3.3)
Switzerland	0 (0.0)	0 (0.0)	1 (3.3)	0 (0.0)	1 (3.3)
**Shiga toxin** **-producing** ***E. coli***	1 (2.4)	3 (7.3)	5 (12.2)	2 (4.9)	11 (26.8)
Seasonality					
Winter (December to February)	0 (0.0)	0 (0.0)	2 (18.2)	2 (18.2)	4 (36.4)
Spring (March to May)	1 (9.1)	3 (27.3)	2 (18.2)	0 (0.0)	6 (54.5)
Summer (June to August)	0 (0.0)	0 (0.0)	1 (9.1)	0 (0.0)	1 (9.1)
Fall (September to November)	0 (0.0)	0 (0.0)	0 (0.0)	0 (0.0)	0 (0.0)
Region of travel					
Latin America/Caribbean	1 (9.1)	3 (27.3)	5 (45.5)	2 (18.2)	11 (100.0)
Mexico	1 (9.1)	3 (27.3)	5 (45.5)	2 (18.2)	11 (100.0)

a Year of outbreak based on the date outbreak was reported to CDC.

b Percentages might not total 100 due to rounding.

c Eight outbreaks involved people reporting travel to more than one country or region.

d Region of travel might not have the same total as the individual listed countries due to some people in the outbreak reporting travel to more than one country prior to illness onset or more than one region or country reported by various people in the outbreak.

### Descriptive analysis

Of the1,066 illnesses, most were nontyphoidal *Salmonella* infections (92.1%, 982/1066). Among people with available demographic information, the median age of ill people with nontyphoidal *Salmonella* infections was 29 years (range: <1–87), 59.2% (553/934) were female, seven (1.4%, 7/516) were hospitalized, and three (0.6%, 3/513) people died (Table 2).

**Table 2.** Demographics of nontyphoidal *Salmonella* and Shiga toxin-producing *Escherichia coli* outbreak-associated ill people including hospitalization and death by year and pathogen—United States, 2017–2020

	2017^a^^,^^b^	2018	2019	2020	Total
Characteristics	*n* (%)	*n* (%)	*n* (%)	*n* (%)	*n* (%)
**Demographic characteristics**					
*Ill people n* ^c^^,^^d^	313 (29.4)	229 (21.5)	441 (41.4)	83 (7.8)	1,066 (100)
**Nontyphoidal** ***Salmonella***	304 (28.5)	206 (19.3)	399 (37.4)	73 (6.8)	982 (92.1)
Age (*n*)					903 (92.0)
≤5	12 (1.3)	17 (1.9)	29 (3.2)	2 (0.2)	60 (6.6)
6–18	36 (4.0)	24 (2.7)	34 (3.8)	11 (1.2)	105 (11.6)
18–34	81 (9.0)	39 (4.3)	75 (8.3)	15 (1.7)	210 (23.3)
34–64	142 (15.7)	80 (8.9)	173 (19.2)	35 (3.9)	430 (47.6)
≥65	30 (3.3)	19 (2.1)	39 (4.3)	10 (1.1)	98 (10.9)
Sex (*n*)					934 (95.1)
Male	122 (13.1)	84 (9.0)	141 (15.1)	34 (3.6)	381 (40.8)
Female	170 (18.2)	112 (12.0)	234 (25.1)	37 (4.0)	553 (59.2)
Hospitalization (*n*)					516 (52.5)
Hospitalized	1 (0.2)	1 (0.2)	1 (0.2)	4 (0.8)	7 (1.4)
Deaths (*n*)					513 (52.2)
Died	1 (0.2)	1 (0.2)	1 (0.2)	0 (0.0)	3 (0.6)
**Shiga toxin** **-producing** ***E. coli***	9 (0.8)	23 (2.2)	42 (3.9)	10 (0.9)	84 (7.9)
Age (*n*)					83 (98.8)
≤5	0 (0.0)	5 (6.0)	5 (6.0)	0 (0.0)	10 (12.0)
6–18	2 (2.4)	3 (3.6)	3 (3.6)	1 (1.2)	9 (10.8)
18–34	5 (6.0)	10 (12.0)	17 (20.5)	3 (3.6)	35 (42.2)
34–64	1 (1.2)	4 (4.8)	15 (18.1)	5 (6.0)	25 (30.1)
≥65	1 (1.2)	1 (1.2)	2 (2.4)	0 (0.0)	4 (4.8)
Sex (*n*)					80 (95.2)
Male	3 (3.8)	7 (8.8)	17 (21.3)	3 (3.8)	30 (37.5)
Female	5 (6.3)	16 (20.0)	23 (28.8)	6 (7.5)	50 (62.5)
Hospitalization (*n*)					62 (73.8)
Hospitalized	1 (1.6)	0 (0.0)	0 (0.0)	0 (0.0)	1 (1.6)
Deaths (*n*)					62 (73.8)
Died	1 (1.6)	0 (0.0)	0 (0.0)	0 (0.0)	1 (1.6)

a Year of illness based on the date of isolate collection.

b Date of isolation collection for 18 ill people in two of the 2017 outbreak investigations was in 2016. Data for these cases have been included in 2017.

c Percentages might not total 100 due to rounding.

d Variables are reported based on information available. Total available data for each variable is noted in the total column. Percentages were calculated without missing data using only the total available data for each variable.

The 11 STEC outbreaks involved 84 (7.9%, 84/1066) ill people. The median age of people with STEC infections was 39 years (range: <1–90) and 50 (62.5%, 50/80) were female. One (1.6%, 1/62) person was hospitalized and one (1.6%, 1/62) person died.

**Figure 3.**
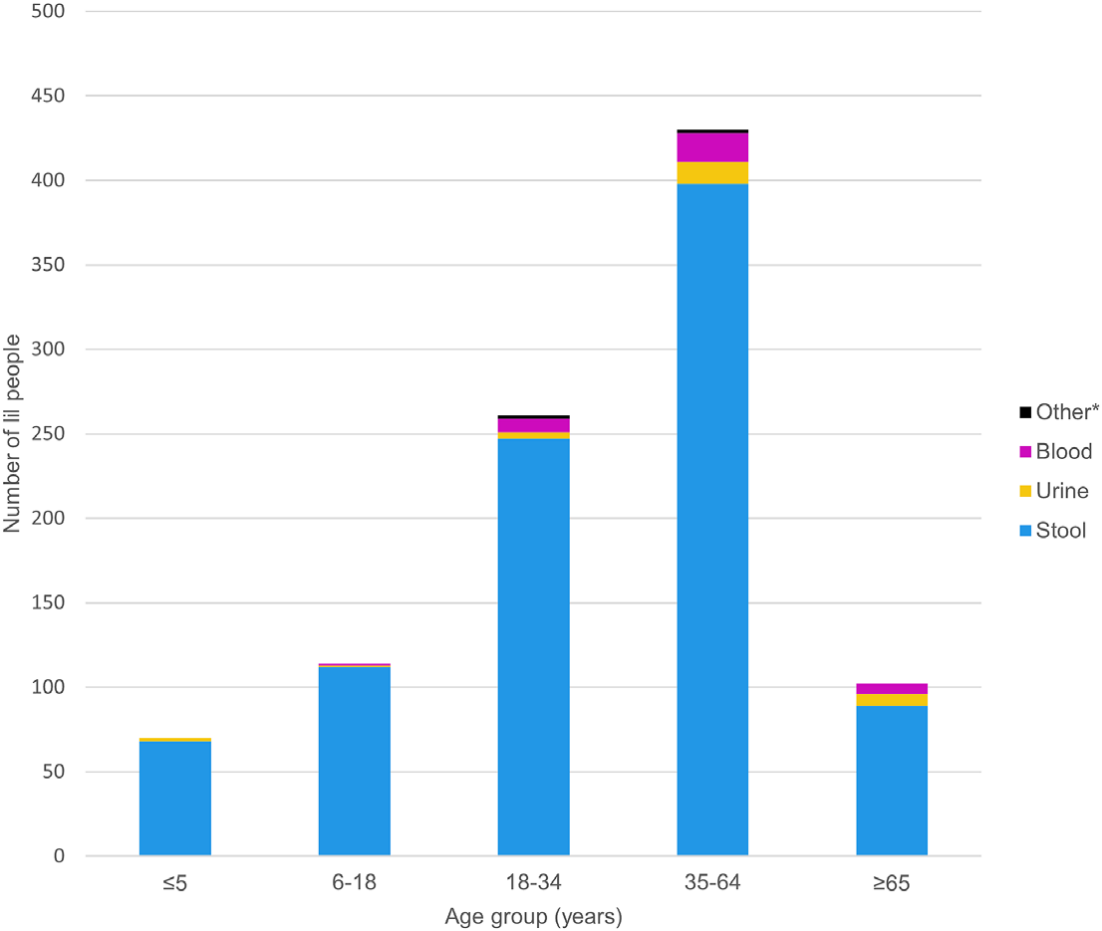
Number of ill people and specimen source in multistate travel-associated outbreaks by age group—United States, 2017–2020. Variables are reported based on information available. Unknown data were considered missing. Information on age and source was available for 977 ill people. *Other sources included skin, wound, gall bladder, abscess, respiratory cerebrospinal fluid, or other tissue or body fluid.

Six (75%, 6/8) of the hospitalized people and all four (100%, 4/4) of the people who died had MDR infections. The median age of people who died was 66 years (range: 17–82): three had *Salmonella* Newport infections and one had an O103:H2 infection. Most patients (93.3%, 984/1055) had *Salmonella* or STEC isolated from a stool sample. Adults older than 65 years had the highest proportion of blood isolates (5.9%, 6/102) (Figure 3).

Of the 631 people with epidemiological information available, 437 (70%) reported international travel. Specifically, 317 (73%) reported travel to Mexico and 81 (19%) to the Dominican Republic. All people (100%, 62/62) with STEC infections reported travel to Mexico.

### Analysis of AR

AR data were available for 982 isolates from the 30 nontyphoidal *Salmonella* and 11 STEC outbreaks (Table 3). Strains from 14 of 30 (47%) nontyphoidal *Salmonella* outbreaks and 3 of 11 (27%) STEC outbreaks were not associated with resistance and were caused by serotypes Enteritidis (6 outbreaks), Typhimurium (2), Muenchen (2), Thompson (1), Concord (1), Newport (1) Strathcona (1), O111 (2), and O123/O186 (1).

**Table 3.** Resistance profiles of multistate nontyphoidal *Salmonella* and Shiga toxin-producing *Escherichia coli* outbreaks including serotype and travel region by year and pathogen—United States, 2017–2020

		Number of ill people in outbreak	Isolates with resistance information	Isolates with any resistance^a^	Isolates with MDR^a^^,^^b^	Isolates with clinically significant resistance^a^^,^^c^^,^^d^	Travel regions			
Year	Serotype of outbreak	*n*	*n*	*n* (%)	*n* (%)	*n* (%)	Latin America/ Caribbean^e^	Canada	Europe^f^	Asia^g^
**Nontyphoidal*****Salmonella***										
2017	Braenderup	10	8	8 (100.0)	0 (0.0)	7 (87.5)	X			
	Enteritidis	12	8	6 (75.0)	0 (0.0)	6 (75.0)	X			X
	Enteritidis	18	12	12 (100.0)	0 (0.0)	3 (25.0)	X			
	Enteritidis	5	2	2 (100.0)	0 (0.0)	2 (100.0)	X			
	Enteritidis	7	7	7 (100.0)	6 (85.7)	7 (100.0)	X			
	Enteritidis	125	100	95 (95.0)	0 (0.0)	95 (95.0)	X			
	Enteritidis	12	11	9 (81.8)	0 (0.0)	9 (81.8)	X			X
	Muenchen	13	11	0 (0.0)	0 (0.0)	0 (0.0)	X			
	Muenchen	24	20	0 (0.0)	0 (0.0)	0 (0.0)	X			
	Newport	53	52	49 (94.2)	49 (94.2)	49 (94.2)	X			
	Typhimurium	7	1	0 (0.0)	0 (0.0)	0 (0.0)	X			
	Typhimurium	18	16	0 (0.0)	0 (0.0)	0 (0.0)	X			
2018	Newport	365	356	306 (86.0)	305 (85.7)	306 (86.0)	X			
	Concord	8	8	0 (0.0)	0 (0.0)	0 (0.0)		X	X	
2019	Enteritidis	13	11	11 (100.0)	1 (9.1)	11 (100.0)	X		X	X
	Enteritidis	21	19	19 (100.0)	0 (0.0)	19 (100.0)	X			
	Enteritidis	25	25	0 (0.0)	0 (0.0)	0 (0.0)	X			
	Enteritidis	57	56	2 (3.6)	0 (0.0)	1 (1.8)	X			
	Enteritidis	23	23	5 (21.7)	0 (0.0)	0 (0.0)	X			
	Newport	8	8	0 (0.0)	0 (0.0)	0 (0.0)	X			
	Strathcona	4	4	0 (0.0)	0 (0.0)	0 (0.0)			X	
	Thompson	47	40	0 (0.0)	0 (0.0)	0 (0.0)	X			
2020	Enteritidis	13	13	0 (0.0)	0 (0.0)	0 (0.0)	X			
	Enteritidis	20	20	0 (0.0)	0 (0.0)	0 (0.0)	X			
	Enteritidis	11	11	4 (36.4)	0 (0.0)	0 (0.0)	X			
	Enteritidis	11	10	0 (0.0)	0 (0.0)	0 (0.0)	X			
	Enteritidis	11	11	11 (100.0)	0 (0.0)	11 (100.0)	X			
	Enteritidis	9	9	0 (0.0)	0 (0.0)	0 (0.0)	X			
	Enteritidis	19	19	5 (26.3)	1 (5.3)	2 (10.5)	X			
	Typhimurium	13	13	13 (100.0)	13 (100.0)	13 (100.0)	X			
**Shiga toxin** **-producing** ***E. coli***										
2017	O103:H2	9	6	6 (100.0)	6 (100.0)	N/A	X			
2018	O111:H8	8	6	6 (100.0)	4 (66.7)	N/A	X			
	O157:H7	6	6	6 (100.0)	6 (100.0)	N/A	X			
	O157:H7	8	9	1 (11.1)	0 (0.0)	N/A	X			
2019	O103:H2	7	6	6 (100.0)	6 (100.0)	N/A	X			
	O111:H8	10	10	9 (90.0)	9 (90.0)	N/A	X			
	O111:H8	12	11	0 (0.0)	0 (0.0)	N/A	X			
	O111:H8	7	7	0 (0.0)	0 (0.0)	N/A	X			
	O111:H8	6	6	5 (83.3)	5 (83.3)	N/A	X			
2020	O123/O186:H2	6	6	0 (0.0)	0 (0.0)	N/A	X			
	O111:H8	5	5	5 (100.0)	1 (20.0)	N/A	X			

N/A, not applicable

a Includes isolates with nonsusceptible interpretation to ciprofloxacin by AST or ≥1 ciprofloxacin resistance mechanism when no AST results were available.

b MDR, resistance to antimicrobials from ≥3 drug classes.

c Clinically significant resistance = resistance to ≥1 antimicrobial recommended for empiric or alternative treatment of salmonellosis (ampicillin, azithromycin, ceftriaxone, ciprofloxacin, or trimethoprim-sulfamethoxazole). No resistance is considered clinically significant for STEC outbreaks because antimicrobials are not recommended to treat STEC infections.

d NARMS testing panel for AST included streptomycin from 2017 to 2019, which was then replaced by colistin in 2020, increasing azithromycin by one dilution.

e Latin America/Caribbean includes patients reporting travel to Aruba, Cuba, Dominican Republic, Jamaica, Mexico, and Caribbean cruise.

f Europe includes patients reporting travel to Europe, Denmark, France, Germany, Italy, Spain, and Switzerland.

g Asia includes patients reporting travel to Asia, Philippines, andThailand.

A total of 16 (53.3%, 16/30) nontyphoidal *Salmonella* and eight (72.7%, 8/11) STEC outbreaks contained ≥10% isolates that conferred resistance to one or more of the drugs tested by NARMS. At least 10% of the isolates in 14 nontyphoidal *Salmonella* outbreaks conferred resistance to one or more of the clinically significant antimicrobials used in human medicine. Of the nontyphoidal *Salmonella* isolates with resistance information, 541/904 (59.8%) were associated with clinically significant resistance. More than half of those isolates, 306/541 (56.6%) represent a single *Salmonella* Newport outbreak (Table 3). Four *Salmonella* outbreaks had ≥10% isolates with MDR, including two caused by serotype Newport; both Newport outbreaks included isolates that showed resistance to clinically significant antimicrobials such as ampicillin, azithromycin, ciprofloxacin, and trimethoprim-sulfamethoxazole as well as other antimicrobials not indicated for clinical use (e.g., chloramphenicol, streptomycin, sulfisoxazole, and tetracycline). The remaining two MDR *Salmonella* outbreaks were caused by serotypes Enteritidis and Typhimurium; 100% (7/7) of the *Salmonella enteritidis* isolates showed resistance to chloramphenicol, ciprofloxacin, nalidixic acid, streptomycin, sulfisoxazole, and tetracycline, and 77% (10/13) of the *Salmonella typhimurium* isolates showed resistance to ampicillin, streptomycin, sulfisoxazole, and tetracycline (Supplementary Table S1). Nearly half (46.7%, 14/30) of the *Salmonella* outbreaks included isolates with clinically significant resistance, including serotype Enteritidis (10 outbreaks), Newport (2), Typhimurium (1), and Braenderup (1).

The majority (64%, 7/11) of the STEC outbreaks had ≥10% of isolates with MDR, specifically resistance to ampicillin, chloramphenicol, streptomycin, sulfisoxazole, tetracycline, ciprofloxacin, nalidixic acid, or kanamycin (Supplementary Table S1). Only one of the MDR STEC outbreaks was caused by the O157 serotype, and the remaining resulted from serotypes O111 (4 outbreaks) and O103 (2 outbreaks). One STEC outbreak included one isolate (11%, 1/9) with resistance to fosfomycin.

## Discussion

The investigation of 41 multistate travel-associated nontyphoidal *Salmonella* and STEC outbreaks over a 4-year period demonstrates that returning US travellers are acquiring infections from travel to international destinations. In the same time frame from 2017 to 2020, the CDC investigated 470 domestic possible and confirmed multistate enteric disease outbreaks caused by *Salmonella*, STEC, and *Listeria monocytogenes* [22]. The median age and sex of people ill from travel-associated infections in this analysis are similar to those of domestically acquired foodborne infections; however, reported hospitalizations and deaths for travel-associated infections is much lower [22]. This might be a result of the ‘healthy traveller effect’, which assumes people who travel may be healthier than those who choose not to travel [23] or people may have sought care abroad. Many enteric bacterial illnesses are attributable to international travel each year; the number of nontyphoidal *Salmonella* and STEC illnesses presented here are likely an underestimation of the true number of illnesses because of self-limiting infections that resolve prior to returning to the United States.

Travel-associated outbreaks were commonly associated with popular international destinations for US travellers. This might account for the high number of people in these outbreaks reporting travel to Mexico prior to illness onset, since Mexico was the most common international destination for US travellers during 2017–2020. December and March are two of the highest travel months to Mexico, which might influence the seasonality of STEC outbreaks noted in the winter and spring [1]. Other factors such as livestock management practices [24] or the growing, harvesting, and availability of food during various times of the year may impact seasonality [25]. The risk of acquiring enteric illness during travel likely varies from country to country based on differences in knowledge and implementation of food safety standards, cold storage and infrastructure challenges, and access to potable water for food vendors [26]. Collaboration with international partners could result in the implementation of public health interventions to improve hygiene practices and food safety standards in hotels and restaurants [27]. Additionally, less availability and use of WGS or other subtyping methods in resource-constrained settings might reduce detection and subsequent efforts to prevent enteric bacterial disease outbreaks [28]. Our findings emphasize that an improved epidemiological understanding of enteric bacterial illnesses linked to international travel might provide insight into developing public health guidance for travellers and healthcare professionals as well as a better understanding of challenges faced by other regions related to preventing enteric illnesses.

Although we identified multiple nontyphoidal *Salmonella* serotypes, Enteritidis, Typhimurium, and Newport were most commonly associated with travel-associated outbreaks. These serotypes appear to be consistently reported in ill people returning from international travel [29]. However, this might be expected as these serotypes are consistently three of the most common serotypes causing domestically acquired outbreak-associated illness in the United States [30]. Outbreaks in some countries are regularly linked to a particular serotype; conversely, some serotypes are consistently associated with travel to a single country. We found that all outbreaks linked to the Dominican Republic and Jamaica were caused by *S. enteritidis* and all *Salmonella* Newport and STEC outbreaks were linked to Mexico. This suggests there might be geographic associations with certain serotypes which could assist with epidemiological investigations and identification of a common source of illness.

AR is a growing global concern, with drug-resistant nontyphoidal *Salmonella* considered a serious public health threat [31]. In our analysis, more than half of all nontyphoidal *Salmonella* infections were associated with clinically significant resistance, compared to 14.0–16.4% for all nontyphoidal *Salmonella* infections tested by NARMS from 2017 to 2020 [32]. This might limit treatment options for the management of infections in returned travellers. Additionally, the resistance among nontyphoidal *Salmonella* and STEC isolates highlights the importance of a global One Health approach to antimicrobial stewardship and the potential selection pressures that might result in the development of resistance in enteric pathogens. Evaluating demographics, food, water, healthcare, animal contact exposures, and travel locations alongside AR data will increase the understanding of the risk of MDR infections for travellers and ways that these concerning bacteria might spread or be introduced into the United States. An improved understanding might support focused public health messaging, prevention strategies, and improved communication with internationalpartners.

The findings in this report are subject to several limitations. First, detailed demographics, travel history, and potential food, water, healthcare, and animal exposures were not available for all people because some people did not respond to requests for information from health departments or provided incomplete information. All information was obtained from patient interviews and might be subject to recall bias. Most patients did not provide detailed travel histories so we were unable to link travel-associated outbreaks to specific exposures (e.g., food, water, or animal contact) in these cases. Lacking an identified exposure limits the actionable information we can provide to country health officials and reduces the ability to provide focused prevention messages. Additionally, data were not available regarding a patient’s reason for travel (e.g., leisure, business, medical, or visiting friends and relatives) and prevention messaging might differ based on travel type. Furthermore, we cannot assume that all illnesses in the outbreaks were linked to international travel. In the absence of a confirmed source of illness in these outbreaks, we used people reporting international travel in the 7 days before illness onset as exposure criteria to define travel-associated outbreaks. These criteria may have resulted in the inclusion of people with domestically acquired infections or exclusion of people with travel-associated infections due to shorter or longer incubation times. For people not reporting international travel prior to illness onset, domestically acquired infections could be occurring as demonstrated in an outbreak which involved both people who consumed a contaminated food product while travelling internationally and those who consumed a different food product contaminated with the same outbreak strain imported to the United States from the same country [33]. Globalization of food systems might result in outbreak-associated illnesses affecting people in more than one country, including local people in countries linked to travel-associated outbreaks.

Second, we did not have AR information for all isolates, especially for outbreaks that were reported beforemid-2019. This might have resulted in missed resistance or inappropriate characterization of the resistance profile of some outbreaks. One reason for missing AR information might be that some public health laboratories might not prioritize WGS of isolates from international travellers because the source of the infection is less likely to require intervention by the state or local health department, thus limiting the ability to detect outbreaks at the national level.

Third, the number and size of travel-associated outbreaks in 2020 might have been impacted by the COVID-19 pandemic: public health capacity to detect, investigate, and report outbreaks of enteric illnesses was limited and travel restrictions were in place throughout parts of the year. People might have been less likely to visit a healthcare professional for mild symptoms and thus less likely to receive a culture necessary to identify a nontyphoidal *Salmonella* or STEC infection. Additionally, public health laboratories might have been less likely to perform WGS on an isolate, which is necessary for linking a patient to an outbreak, as a result of limited or diverted laboratory capacity in response to the COVID-19 pandemic. We chose to include outbreaks from 2020 in the analysis given the number of outbreaks early and late in the year, times possibly less affected by travel bans and restrictions.

Lastly, we are unable to compare or evaluate the frequency and characteristics of single-state travel-associated outbreaks. These data are not available since CDC does not routinely investigate single-state outbreaks, and outbreaks linked to international travel are not reportable to the National Outbreak Reporting System.

Enteric bacterial infections and subsequent outbreaks are linked to international travel each year, and many of these illnesses exhibited AR. Education and public health messaging and pretravel consultations [34] could reduce enteric disease illness in people who travel. Investigating illness outbreaks among returned travellers could provide the opportunity to prevent additional illness and spread of MDR organisms domestically and internationally.

## Supporting information

Habrun et al. supplementary material Habrun et al. supplementary material

## Data Availability

If interested in accessing data or other materials related to this manuscript, readers may contact the corresponding author.

## Supplementary material

For supplementary material accompanying this paper visit http://doi.org/10.1017/S0950268823002017. click here to view supplementary material
